# Subacute Pain as a Predictor of Long-Term Pain Following Orthopedic Surgery: An Australian Prospective 12 Month Observational Cohort Study: Erratum

**DOI:** 10.1097/01.md.0000499794.09212.7e

**Published:** 2015-10-30

In the article “Subacute Pain as a Predictor of Long-Term Pain Following Orthopedic Surgery: An Australian Prospective 12 Month Observational Cohort Study”,^1^ which appeared in Volume 94, Issue 36 of *Medicine*, several variables in Table 1 were incorrectly indented when they should have been left justified. The article has since been corrected online.
